# Leveraging Electron Beam (eBeam) Technology for Advancing the Development of Inactivated Vaccines

**DOI:** 10.3390/vaccines13020179

**Published:** 2025-02-13

**Authors:** Ruvindu Perera, Suresh D. Pillai, Adnan Alrubaye, Palmy Jesudhasan

**Affiliations:** 1 Cell and Molecular Biology Program, University of Arkansas, Fayetteville, AR 72701, USA; rperera@uark.edu; 2 National Center for Electron Beam Research, Texas A&M University, College Station, TX 77840, USA; suresh.pillai@ag.tamu.edu; 3 Center of Excellence for Poultry Science, University of Arkansas, Fayetteville, AR 72701, USA; 4 Poultry Production and Product Safety Research Unit, USDA-ARS, Fayetteville, AR 72701, USA

**Keywords:** electron beam, eBeam, inactivated vaccines, bacteria, virus, radiation

## Abstract

This review provides an overview of electron beam (eBeam) technology and its applications across a wide variety of disciplines. More importantly, it discusses this technology’s advantages and its benefits in developing inactivated vaccines. eBeam technology is currently being used all around the world for a variety of industrial applications, extending from food pasteurization to the cross-linking of polymers in the wire and cable industries. It is a successful emerging alternative for developing vaccines against bacterial, protozoan, and viral pathogens. This review includes a descriptive account of the mechanism of action of eBeam and how this technology achieves the complete inactivation of pathogens while retaining the integrity of their surface epitopes. This unique advantage is crucial for the production of efficacious vaccines. This review provides a detailed account of the usage of eBeam technology for developing vaccines to protect a multitude of hosts against a wide range of pathogens. eBeam-inactivated vaccines are advantageous over live vaccines, RNA/subunit vaccines, and chemically inactivated vaccines mainly due to the complete inactivation of pathogens, and the presence of intact, highly antigenic epitopes. To conclude, this article descriptively highlights eBeam technology’s advantages over other means of vaccine development.

## 1. Types of Radiation

Radiation is defined in physics as the energy emitted from a body or source that is transmitted through an intervening medium or space and absorbed by another body [1]. Radiation technology is currently used for numerous applications in diverse fields. Radiation is categorized based on its wavelength and energy into two main types. Non-ionizing radiation is long-wavelength/low-energy radiation, which includes low-energy UV and other waveforms of higher wavelength. Ionizing radiation is short-wavelength/high-energy radiation, which includes high-energy UV and waveforms of shorter wavelength (i.e., gamma and X-rays) [1,2]. Ionizing radiation, as defined, is capable of ionizing events in the materials it encounters in that it ionizes (removes electrons from) the atoms in the materials [3,4,5,6,7]. Non-ionizing radiation, as the term implies, is incapable of ionization events [2,4]. Frequently used types of ionizing radiation include gamma (photon-based), electron beam (eBeam: electron-based), and X-ray (photon-based) [3,8]. Electron beam, despite being ionizing radiation, is not part of the electromagnetic spectrum because these are discrete particles with a known mass and energy. Ionizing radiation as used for industrial applications is obtained from radioisotopes (such as cobalt-60 and cesium-137 for gamma). Electron beam and X-ray are ionizing radiation that is produced using industrial equipment called accelerators. Different types of accelerators are commercially available for different industrial applications. Generally speaking, accelerators are available for low-energy eBeam (LEEB), low-energy X-ray (LEEX), medium-energy eBeam (MEEB), medium-energy X-ray (MEEX), high-energy eBeam (HEEB), and high-energy X-ray (HEEX). What is considered “low”, “medium”, and “high” are arbitrary, but in general, eBeam and X-ray with energies less than 1 MeV are considered low-energy, accelerators generating electrons and X-rays in the 1–4 MeV range are considered medium-energy, and radiation between 4 and 10 MeV is considered high-energy [8].

Each type of ionizing technology has its own advantages and disadvantages. Gamma radiation from cobalt-60 is made up of energetic photons between 1.17 MeV and 1.33 MeV. Since these are uncharged photons, they do not interact with materials as much as the particulate charged electrons, and therefore, gamma radiation penetrates more [8,9]. However, gamma irradiation has several downsides. This technology utilizes radioactive isotopes, and therefore, the radiation cannot be switched on/off as and when needed. Also, since this technology relies on radioactive material, there are serious security issues associated with its use. Accidental exposure to this radioactive material is a major occupational hazard in such facilities [10,11]. Additionally, the dose rate (amount of dose delivered per unit time) is significantly less than that of eBeam or X-rays, and therefore, from an industrial operations point of view, it is not as efficient as either eBeam or X-ray technologies. Furthermore, the disposal of radioactive materials involves significant expenses [3,12]. Most importantly, the cost and shortage of radioactive isotope cobalt-60 are serious issues that have plagued this technology [13,14]. X-ray technology is based on photons generated by the bombardments of accelerator-generated energetic electrons onto a highly dense metal (e.g., tantalum or tungsten) [3]. X-ray irradiation has certain advantages over gamma, such as not needing a radioactive source and being able to switch on/off depending on use [3,15]. However, X-ray technology is hampered by power losses due to the low conversion efficiency of eBeam energy to X-ray energy (most of the electron kinetic energy is converted to heat) [16]. The eBeam process is theoretically the most cost-effective approach of utilizing ionizing technology. However, the cost of acquiring either HEEB eBeam or X-ray technologies is relatively high, given that these types of equipment are sophisticated pieces of machinery. There are several reference books that detail the underlying principles of eBeam, X-ray, and gamma technologies [8,17]. The cost effectiveness of eBeam compared to X-ray production is also driven by its higher conversion efficiency [18] (electricity → eBeam). Electron acceleration, depending on whether it is LEEB or MEEB, or HEEB as mentioned above, yields up to 10 MeV (Mega electron volts) of energy [3,17]. In addition to possessing the advantages of X-ray mentioned above, eBeam is safe to use under aqueous conditions. eBeam does not generate toxic compounds under aqueous conditions but generates high dose rates (minimizing heat generation) [9].

Furthermore, eBeam exposure is substantially time-conservative due to the ability to generate higher dose rates (kGy/s) compared to gamma (10^4^-fold) and X-ray (10^3^-fold) [19]. The limitation of eBeam technology is its low penetration power, which is far inferior to that of X-ray. However, this can be mitigated by optimizing the substrate’s size, dimensions, and specific density prior to eBeam processing [12]. eBeam irradiation has gained popularity over recent years owing to its undeniable benefits, safety, and feasibility. Further, ionizing radiation technology is an approved means of intervention by the U.S. Food and Drug Administration (FDA) and U.S. Department of Agriculture, Food Safety and Inspection Service (USDA-FSIS) as an alternative to chemical or heat treatments for reducing foodborne pathogens and adulterants in meat products and spices [20,21,22]. This review focuses on eBeam in various applications, primarily in vaccine development.

## 2. Applications of Electron Beam Technology

### 2.1. A Wholistic View of eBeam-Related Applications

The diverse applications of electron beam technology (EBT) are summarized in Table 1.

**Agriculture:** When the field of agriculture is considered, EBT has proven to play a vital role in decontaminating vegetables, fruits, meat, and meat products while preserving their taste and quality. EBT significantly reduces microbial load while having a low impact on the nutrient content [23]. eBeam has also been shown to increase the shelf-life of crops, fruits, and vegetables by controlling their germination and ripening rate [23,24]. Furthermore, microbial decontamination [23,24,25], disinfestation of insects [23,24,25], elimination of anti-nutritional compounds [23,24], inhibition of germination [23,24], and enhancement in functionality in a variety of cereals [23,24] are among the adaptations of EBT for food safety.

**Medicine:** In the field of medicine [26], studies have depicted the potential of EBT to treat superficial lesions [27] and tumors of the lungs [28], prostrate [29,30], brain, head, and neck [31]. Moreover, continuous eBeam sterilization processing is used to efficiently sterilize medical equipment and vessel surfaces [32,33].

**Environment:** EBT is used in wastewater treatment in different ways. EBT, in synchrony with ozone treatment, coagulation, and activated sludge techniques for textile wastewater treatment, has been shown to decrease discoloration, turbidity, COD, BOD, pH, and wastewater conductivity more effectively. Additionally, eBeam treatment of municipal wastewater reduces BOD, COD, and coliforms (more than 90%) efficaciously [34,35], making eBeam technology a potential supplement when other methods alone are inefficient. Furthermore, cross-linking of Laccase (enzyme from aquatic ascomycete, *Phoma* sp.) to PVDF (polyvinylidene fluoride) membranes by EBT has been shown to immobilize and remarkably increase the functional stability of Laccase in wastewater, thereby increasing the efficiency of the removal of pharmaceutically active compounds therein [36]. EBT also obtains >80% degradation and decomposition of Hydroxychloroquine (HCQ) in wastewater, which is an oxidative hemotoxin that causes complications in skin and hair pigmentation and ocular effects [37]. Additionally, treating industrial effluents with EBT results in the degradation of aromatic organic compounds, trihalomethanes, PCE, and TCE in industrial effluents and renders them less toxic [38,39].

**Polymer Industry and Cross-Linking:** EBT is used for cross-linking molecular chains and the scission of polymer chains in polymeric materials [40]. The textile industry uses these properties for surface modifications of fabric; EBT removes impurities and poor boundary layers on the surfaces of fabrics. eBeam processing also changes surface characterization and topography [41,42]. Low-energy electron accelerators are used in coating machines, printing presses, laminating machines, and others. Medium-energy electron accelerators are used to process thick plastic and rubber sheets, wire and cable insulation, plastic tubes, and fiber-reinforced composites, and high-energy EBT is used for tuning semiconductors [43]

**Vaccine Development against Microbial Pathogens:** EBT is used in vaccine developments and explained further in Section 2.2 and Section 3 below.

**Other Industrial Applications:** The cross-linking and scission of chemical compounds and polymers upon exposure to EBT are manipulated and applied for various applications such as (a) the room-temperature synthesis of graphene quantum dots and their application in cell imaging [44]; (b) enhancing optical power and clamping of Al-doped ZnO thin films so that they act as an efficient optical limiter for photonic device applications [45]; (c) the controllable reduction of graphene oxide to optimize its applications in ion sieves, energy storage, biomaterials, conductive materials, and water treatment [46]; (d) improving the ionic conductivity of polymer electrolytes and augmenting their application in lithium-ion batteries for various electronic appliances [47]; (e) the usage of EBT to coat the surface of cotton with a N-halamine precursor [3-(3′-acrylicacidpropylester)-5,5-dimethylhydantoin] to enhance the antimicrobial properties and stability of cotton to be used in the healthcare field [48]; and (f) EBT-mediated reduction in molecular weight and crystallinity of microcrystalline cellulose, accordingly improving the feasibility of its use as a raw material for the production of sugars and ethanol [42].

**Table 1:** Summary of applications of eBeam in various fields and its corresponding involvement.

Field of Application	Involvement of eBeam Technology	References
Agriculture	Decontamination of vegetables, fruits, meat, and meat products	[23]
	Increasing the shelf-life of crops, fruits, and vegetables	[23,24]
	Microbial decontamination, disinfestation of insects, elimination of anti-nutritional compounds, cereal germination inhibition, and enhancing functionality	[8,23,24,25]
Medicine	Treating superficial lesions and tumors of the lungs, prostate, brain, head, and neck	[26,27,28,29,30,31]
	Sterilizing medical equipment and vessel surfaces	[32,33]
	Enhancing the antimicrobial properties and stability of cotton for sterilization	[48]
Environment	Increasing the efficiency of ozone treatment, coagulation, and activated sludge techniques	[34,35]
	Stabilizing the Laccase enzyme to increase its efficiency in wastewater purification	[36]
	Reduction of graphene oxide for use in wastewater treatment	[46]
	Degradation and decomposition of Hydroxychloroquine	[37]
	Degradation of aromatic organic compounds, trihalomethanes, PCE, and TCE in industrial effluent (decreasing toxicity)	[38,39]
Polymer Industry and Cross-Linking	Surface modification of fabrics	[40,41,42]
	Coating machines, printing presses, and laminating machines (low-energy eBeam)	[43]
	Processing thick plastic and rubber sheets, wire and cable insulation, plastic tubes, and fiber-reinforced composites (medium-energy eBeam)	
	Tuning semiconductors (high-energy eBeam)	
Other Industrial Applications	Room-temperature synthesis of graphene quantum dots	[44]
	Increasing the optical limiting efficiency of Al-doped ZnO	[45]
	Controllable reduction of graphene oxide to optimize its applications in ion sieves, energy storage, biomaterials, and conductive materials	[46]
	Improving the ionic conductivity of polymer electrolytes to increase the performance of lithium-ion batteries	[47]
	Processing of cellulose and optimizing its usability as a raw material to produce sugar and ethanol	[42]

### 2.2. Application of eBeam in Vaccine Preparation

A relatively recent application of the EBT is vaccine development. The purpose of any vaccine is to generate immunity against microbial pathogens. The mechanism by which eBeam vaccines carry out their function is as follows. The antimicrobial activity of eBeam occurs via direct and indirect effects, and the net effect is microbial inactivation [3,12]. The direct effects result from the non-specific collision of the highly energetic electrons with the microorganisms. These collisions cause ionizations of the macromolecules within the microbial cells. The absorbed dose, state of the microbial cell, age of the microbial cell, and the matrix in which it is suspended will finally dictate the inactivation levels.

**Figure 1:**
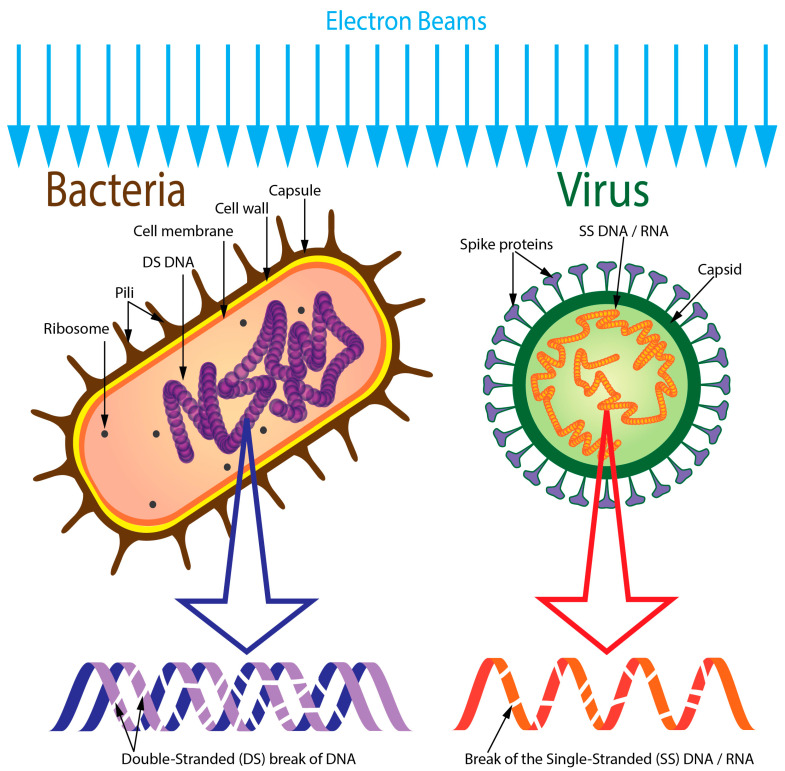
Schematic of microbial inactivation by eBeam via DNA/RNA disintegration. Abbreviations: DNA—deoxyribonucleic acid, RNA—ribonucleic acid, SS—single-stranded, DS—double-stranded. This figure diagrammatically represents eBeam’s effect of causing irreparable breaks in the double-stranded polynucleotide chains of DNA and the single-stranded polynucleotide chain of RNA, thereby inhibiting the ability of microbes to replicate.

As the genetic material (DNA/RNA) is the largest macromolecule within the cells, it is subjected to direct ionization events as the principal target. The phosphodiester bonds of the DNA backbone and the hydrogen bonds between the nitrogenous base pairs (G–C and T–A bonds) are cleaved because of the ionization events. While single-stranded breaks are repairable to a certain extent, extensive double-stranded breaks are hardly repairable. Exposure of a hypothetical genome size of 3.5 million base pairs to a dose of 1 kGy (kilo Gray) would generate approximately 200 single-stranded and 14 double-stranded breaks [3,17,49]. This level of DNA disintegration renders the cell incapable of repairing the DNA damage, and consequently, the cells lose their ability to replicate. This inactivation, resulting in the cell’s ability to divide, is termed cellular reproductive death [3,12]. Figure 1 diagrammatically explains the inactivation of various microorganisms (e.g., bacteria) with double-stranded DNA as their genetic material. Direct ionization by eBeam can also completely inactivate viruses with single-stranded DNA/RNA as their genetic material (Figure 1). Liu et al., 2022 [50], illustrate the mechanism of eBeam action on the inactivation of the Porcine Epidemic Diarrhea Virus (PEDV; a single-stranded RNA virus) via single-stranded RNA disintegration. Direct damage attributable to chemical-bond cleavage is a rapid process, with an estimated completion time of 10^−14^ to 10^−12^ s after eBeam exposure. Therefore, EBT can generate large titers of inactivated cells that could be used as inactivated vaccines.

**Figure 2:**
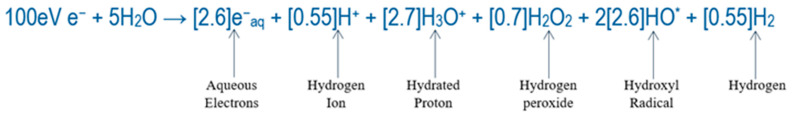
Resultant radiolytic species formed from 100 eV of energetic electrons interacting with water molecules.

The indirect effects of EBT, on the other hand, are due to the interactions of energetic electrons with water molecules, resulting in the formation of highly reactive but extremely short-lived radiolytic species such as aqueous electrons, hydrogen ions, hydrated protons, hydrogen peroxide, hydroxyl radicals, and hydrogen [3,12,17,51]. The equation below (Figure 2) shows the number of species formed when 100 eV of energetic electrons interact with water molecules [3].

The energetic electrons in eBeam interact with the water molecules within the cell, creating a diverse array of highly reactive but short-lived radiolytic (free radical) species such as hydroxyl radicals, hydrogen peroxide, hydrogen, hydrated electrons, and hydrated protons [3]. The highly reactive free radicals seek stability by forming stable products, combining with each other or Oxygen and producing oxidizing agents. Hydroxyl radicals (*OH) significantly damage molecules in the immediate surroundings [52]. Superoxide radicals (O_2−_^∗^) are hypothesized to accumulate within a microbial cell and cause severe damage to proteins like enzymes with exposed iron–sulfur clusters [53,54]. They also react with cellular endogenous nitric oxide species, producing reactive nitrogen species (RNS) that promote DNA damage. Some examples of such RNS are peroxynitrite anions (ONOO^−^), nitrogen dioxide (NO_2_^∗^), and dinitrogen trioxide (N_2_O_3_) [55]. DNA damage restricts further protein synthesis and, thus, the functionality of the microbes. In one picosecond (10^−12^ s), superoxide and hydrogen peroxide radicals are formed. Their reactions are hypothesized to be complete after about 1 millisecond of exposure to EBT [3,56,57]. These oxidizing agents and free radicals non-specifically ‘attack’ or react with the various cellular molecules within the microbial cell. Several studies have proven that exposure of *Clostridium perfringens* up to a 10 kGy dose of EBT [58], exposure of *Salmonella* Typhimurium [59], and *Escherichia coli* [60] up to a 7 kGy dose of EBT, and exposure of *Salmonella* Enteritidis up to a 2.5 kGy dose of EBT [61] effectively inactivated the cells by damaging nucleic acids. However, this preserved the integrity of the cell membrane while leaving the antigenic epitopes unharmed. Cell membrane integrity was confirmed using fluorescence microscopy [58,59,60,61] and electron microscopy [61]. Therefore, it is safe to assume that under well-optimized doses of eBeam, the direct effects are prominent over indirect effects, so the cell membrane is unscathed.

Monte Carlo simulations are used to predict and understand the deposition of a dose within and on a particular object [62,63]. Feng et al., 2020 [64], illustrate the activity of EBT within the irradiated novel coronavirus (COVID-19) capsid via a Monte Carlo numerical simulation. Notably, under both point (passing a narrow beam (string) of electrons through a certain point of the object) and uniform (exposing the whole object uniformly to a wider beam of electrons) irradiation, the activity of eBeam is concentrated at the interior of the capsid (hence disintegrating the RNA). Notably, the activity is minimal at the periphery (thereby maintaining the intact integrity of the surface epitopes). This proves that well-optimized EBT can fully inactivate pathogens while maintaining their surface-structural integrity. The above evidence further highlights why eBeam is an ideal technology for vaccine development. Intact epitopes on the surface of the inactivated pathogen closely resemble the actual pathogen, thus promoting host immunogenicity against the injected, eBeam-inactivated pathogen.

## 3. Application of EBT to Control Pathogens

The efficacy of eBeam technology for the inactivation/control of various bacterial, protozoan, and viral pathogens for different applications has been studied extensively. A very brief review of these studies is presented below. The use of eBeam technology to control bacterial, viral and protozoan parasites are summarized in Table 2, Table 3 and Table 4, respectively.

### 3.1. Control of Bacteria

As explained by Tahergorabi et al., 2012 [12], tomatoes of varying acidity were subjected to eBeam at 0 (control), 0.5, 1.0, 1.5, 2.0, and 2.5 kGy to determine the efficacy against acid-resistant *S.* Montevideo [65]. Significant reductions (up to 2.26-log CFU/g) were recorded in samples exposed to doses greater than 1.5 kGy. The D_10_ values (dose required for achieving 90% reduction [59]) for inactivating *S.* Montevideo in tomatoes were relatively high (1.07–1.50) kGy, and it was concluded that the reasons behind this elevated resistance could be due to a variety of factors including the protective effect of the tomato matrix, the inherent resistance of the acid-resistant bacterial strain and other hitherto-unidentified factors. Moreover, no difference was recorded between the reductions in *S.* Montevideo or *S.* Agona (rifampin-resistant strain) when exposed to eBeam doses of 0.7 and 0.95 kGy when compared to the control (0 kGy), confirming the necessity of at least an eBeam dose of 0.7 kGy to control *Salmonella* in fresh-cut tomatoes [66]. Furthermore, a 3.4- and 4-log reduction in rifampin-resistant *Salmonella* resulted in baby spinach on exposure to eBeam doses of 0.4 and 0.7 kGy, respectively, suggesting that a low-dose eBeam is a viable antimicrobial strategy for minimizing *Salmonella* without product damage [67]. In peanut butter, *S.* Tennessee and *S.* Typhimurium were effectively reduced by EBT [68], whereas the exposed bacterial cells did not resuscitate over a 14-day storage period [69] (hence validating the longevity of bacterial inactivation by eBeam). Fresh eggs, being a popular source of *Salmonella*, resulted in a 5-log reduction in *S.* Enteritidis, *S.* Typhimurium, and *S.* Senftenberg when exposed to 1.5 kGy followed by heat treatment for 3.5 min at 27.7 °C and also when exposed to 0.5 kGy followed by 59.3 °C/3.5 min heat treatment [70], suggesting an optimized protocol for the pasteurization of whole eggs.

### 3.2. Bacterial Inactivation for Bacterial Vaccine Development

As described by Kogut et al., 2012 [71], high-energy eBeam-irradiated (at 2.5 kGy) *Salmonella* Typhimurium (ST) vaccination administered to 18-day chicken embryos significantly reduced ST cecal colonization in the vaccine-treated group (0.95 ± 0.23-log CFU/g of cecal content) as opposed to the positive control (3.48 ± 0.59-log CFU/g)—a 3-log reduction, 5 days after challenge (oral gavage) with the same strain of ST. Furthermore, EBT-treated ST vaccination had a 7-day long, statistically significant heterophil priming and oxidative burst enhancement, with a significantly higher mean fluorescence value (15,857) for phorbol-A-myristate-13-acetate; 2.0 µg/mL (PMA, Sigma-Aldrich, St. Louis, MO, USA) stimulation as opposed to controls (8774.83). Furthermore, heterophil degranulation and bacterial killing mechanisms were intensified in the eBeam-ST vaccine-treated birds, with a significantly higher level of degranulation of β-glucuronidase (118.0) when stimulated with Opsonized ST (OpST), as opposed to the OpST-stimulated controls (88.0).

Bordin et al., 2014 [72], detailed an attempt at eBeam-irradiated vaccine development against *Rhodococcus equi* in neonatal foals. The eBeam (10 Mega electron Volts (MeV)) doses necessary to prevent the replication of *R. equi* were determined as 4 kGy and 5 kGy at 1 × 10^8^ and 1 × 10^9^ CFU/mL titers, respectively. Similar D_10_ values (approximately 0.505 kGy) were obtained for the two pathogen concentrations. Transmission electron microscopy confirmed the preservation of the integrity of the *R. equi* cell membrane after eBeam exposure. Furthermore, eBeam-irradiated vaccine treatment had significantly elevated the IFN-gamma production in peripheral blood mononuclear cells (PMBCs), and the total IgA concentration in nasopharyngeal samples of foals, illustrating the capability of eBeam vaccination to elicit better cell and humoral immune responses against *R. equi*.

Jesudhasan et al., 2015 [61], described a study on the control of *S.* Enteritidis (SE) in 50-week-old single-comb white leghorn molting hens using an eBeam-irradiated vaccine, where the eBeam (10 MeV, 18 kW) dose utilized to irradiate SE was 2.5. kGy. Live/dead bacterial viability staining (BacLight^TM^ bacterial viability stain, Invitrogen, Waltham, MA) followed by fluorescent microscopy revealed only a minute percentage of dead (red-stained) as opposed to living (green-stained) cells. TEM and SEM images depicted similar cell membrane structure and integrity, along with the presence of membrane blebs or micro-vesicles on the cell surfaces in both eBeam-treated and untreated cells. The eBeam-treated cells had not been resuscitated in culture media for up to 4 weeks when incubated at either 37 °C or room temperature. While neither abnormal clinical signs nor morbidity was observed in both the vaccinated and unvaccinated birds, the eBeam-vaccinated group showed significantly lower SE colonization in the ceca, liver, spleen, and ovaries compared to the unvaccinated group. The mean SE enumeration in cecal contents showed a 4-log reduction in the vaccinated group (1.46 ± 0.39-log CFU/g; significantly lower) as opposed to the unvaccinated group (5.32 ± 0.32-log CFU/g). Furthermore, the serum IgG against SE-Lipopolysaccharide had also increased significantly after vaccination and even after the SE challenge (oral gavage of SE) in the vaccinated group compared to the unvaccinated group. The SE vaccine study was the first to prove that eBeam can be used for vaccine development (Patent # US8173139B1) [61].

A study by Fertey et al., 2020 [73], suggests the inactivation of *Rodentibacter pneumotropicus* with low-energy electron beam (LEEB) as a fast and efficient method for generating bacterial vaccines with increased efficacy using 6–8-week-old female BALB/c mice as a model. While growth was detectable after eBeam exposure at 10 kGy (200 kilo electron Volts (keV)), complete inactivation was achieved upon exposure to 20 and 30 kGy. It is extremely important to pay close attention to the dosimetry with low-energy eBeam sources since the dosimetry for LEEB is still very much in its infancy. Importantly, it was discovered that upon exposure to polyclonal serums of previously infected mice via ELISA techniques, eBeam-exposed *R. pneumotropicus* were 100% recognized as opposed to formaldehyde-inactivated (Thermo Scientific, Waltham, MA, USA) *R. pneumotropicus,* which were only 74.7% recognized, depicting better antigenic preservation via eBeam after treatment compared to formaldehyde. Additionally, eBeam inactivation was much more time-conserving (10–15 min) than formaldehyde inactivation treatment (24 h). Significantly higher *R. pneumotropicus*-specific IgG generation was observed in the mice vaccinated with eBeam-treated bacteria (20 kGy) than those vaccinated with formaldehyde-treated bacteria (0.5% formaldehyde) and negative control. Vaccinated mice depicted no clinical score above 1 after challenge with a sub-lethal dose of 6 × 10^6^ CFU *R. pneumotropicus.* However, following the euthanization of the mice, it was realized that no cultivatable bacteria were detectable in the lung tissue of eBeam-vaccinated mice (statistically significant), as opposed to 9 × 10^2^ CFU/g in the formaldehyde-vaccinated group and 7 × 10^4^ CFU/g in the unvaccinated group. Moreover, upon analysis of the LPS functionality via NIH3T3 (ATCC^®^ Number: CRL-1658^™^) reporter cells, it was discerned that eBeam inactivation retained superior *R. pneumotropicus* LPS integrity in contrast to chemical inactivation. Therefore, the above study reinforces the hypothesis that EBT is an excellent alternative to formaldehyde treatment currently utilized commercially in vaccine development.

As Jesudhasan et al., 2021 [58], illustrated, EBT has proven effective in generating chicken vaccines against *Clostridium perfringens* (CP). Eighteen-day-old embryos were vaccinated in ovo with eBeam (10 MeV, 18 kW)-exposed CP at a dose of 10 kGy (based on the D_10_ values for the three CP strains considered, ranging from 0.83 to 1.14 kGy for typical overnight cultures of 10^8^ CFU/mL). It was determined that the cell membrane structure and integrity, metabolic activity, and ultrastructure remained intact between eBeam-treated CP and non-treated (live) CP via LIVE/Dead^®^ BacLight™-stained (ThermoFisher, Waltham, MA, USA) fluorescence measurements, AlamarBlue (ThermoFisher, Waltham, MA, USA) assays, and TEM and SEM imaging, respectively. The surface antigens/epitopes were intact. These studies highlight that an eBeam vaccine possesses the safety of a killed vaccine (as multiplication is prevented due to the result of DNA shredding) but retains the immunogenic potential of a live vaccine. Furthermore, the eBeam-exposed CP cells could not resuscitate in nutrient-rich conditions when incubated anaerobically at 37 °C overnight or at room temperature for up to 4 weeks or in vivo. No significant clinical signs, weight loss, morbidity, or death was observed in vaccinated and unvaccinated birds. However, based on the enumeration of intestinal CP colonization after two rounds of challenges, 60%, 70%, and 100% reductions in CP colonization were reported in the three groups of birds immunized with the three different strains of CP. Finally, a significant increase in serum Ig-Y of all vaccinated birds was observed compared to the negative control (unvaccinated, unchallenged) and positive control (unvaccinated, challenged) birds, confirming the superiority of the efficacy of EBT for vaccine development against CP in chicken.

The efficacy of EBT for inactivating a Gram-positive bacterium (*Bacillus cereus*) and a Gram-negative bacterium (*Escherichia coli*) has been determined [74]. While an eBeam (200 keV) dose of 2.2 kGy was adequate to successfully inactivate a mean *E. coli* titer of 1.67 × 10^7^ CFU/mL, a mean titer of 4.33 × 10^6^ CFU/mL *B. cereus* required 33 kGy, highlighting that the dosimetry with LEEB should be carefully understood. Analysis of the antigenic structural component conservation via ELISAs with polyclonal sera resulted in 73.3–83.5% conservation for *B. cereus* and 83.7–91.4% for *E. coli* portraying the maintenance of acceptable membrane and antigenic epitope intactness after eBeam exposure.

Praveen et al., 2021 [59], have assessed the microbiological correlates and immunostimulatory potential of eBeam-inactivated *Salmonella* Typhimurium (ST) in detail. Having determined the D_10_ value for ST as 0.19 kGy, it was decided that complete inactivation was achieved at 7 kGy (10 MeV, 15 kW) based on the following observations: no growth in culture media for 10 days either at 37 °C or room temperature (RT), absence of *Salmonella* in mice shedding for 14 days post-inoculation (oral gavage), and absence of viable and culturable *Salmonella* in the liver, spleen, cecum and mesenteric lymph node tissues up to 14 days post-inoculation. TEM, SEM, and LIVE/Dead^®^ BacLight™-stained (ThermoFisher, Waltham, MA, USA) fluorescence microscopy depicted the maintenance of intact cell membranes upon EBT. The immunoreactivity profiles (detected via immunoblotting) of eBeam-treated ST (EBST) were highly similar to those of live ST and were unaltered by stressors such as storage at low temperatures (RT, 4 °C, −20 °C) and lyophilized conditions. Interestingly, EBST showed even higher metabolic activity compared to live ST, as indicated by the Alamar Blue^®^ (ThermoFisher, Waltham, MA, USA) assay. EBST had stimulated murine dendritic cell maturation with increased expressions of MHC-II, CD40, CD80, CD86, and TNFα, similar to levels expressed by commercial live attenuated vaccines. Moreover, even after a 6-month storage period at various conditions (room temperature, 4 °C, −20 °C, and lyophilization), EBST successfully stimulated the dendritic cells of mice. The expression levels of CD40, MHC-II, CD80, and CD86 brought up by 6-month-old EBST stimulation (irrespective of the storage condition) were upregulated and on par with the level of live ST stimulation. Therefore, this study further supports the hypothesis that an eBeam-inactivated ST vaccine possesses the safety of a “killed” vaccine while retaining the immunogenicity of a “live attenuated” vaccine.

Chang et al., 2023 [75], describe an attempt to develop an adjuvant using eBeam-treated α-Hemolysin (Hla), a pore-forming toxin produced by *Staphylococcus aureus*. For this study, the Hla protein was purified into 10 mmol/L PBS and directly exposed to a high-energy (10 MeV/20 kW) eBeam dose range of 0 to 4 kGy. It was detected (via SDS-PAGE, Western blot, circular dichroism spectroscopy, and Fourier transform infrared spectroscopy analyses) that the secondary (β-pleated sheet) structure and, hence, the spatial conformation of the Hla protein were significantly altered by exposure to 2–4 kGy of eBeam. It was confirmed via a series of in vitro and in vivo experiments that Hla’s ability to disrupt intestinal and skin tissue epithelial cells (of mice) was altered via eBeam treatment. For eBeam-treated Hla, 0, 1, and 2 kGy doses depicted varying degrees of epithelial cell destruction with sporadic loss of nuclear staining, development of subepithelial gaps in villi, blunting of villi, destruction of the crypts, and overall tissue structure. It was observed that the severity of the damage was indirectly proportional to the eBeam dose used to treat Hla, where 0 and 1 kGy treated Hla caused severe damage, and 2 kGy treated Hla caused less severe damage to the epithelial cells. Damage caused by the 4 kGy treated Hla was barely visible, and the structure of epithelial tissue did not differ significantly from the control group. Finally, it was also verified that exposure to 4 kGy disrupts the ability of Hla to interact with its high-affinity receptor (ADAM10) of epithelial cells. Therefore, it was concluded that eBeam-treated Hla is suitable for use as an immune adjuvant, and further studies are required to validate its potential in vaccine development against *S. aureus*.

### 3.3. Control of Viruses

Fresh vegetables are a popular source of hepatitis A virus (HAV)-related infections, leading to various human foodborne illnesses. Son et al., 2023 [81], report an attempt to use EBT in synergy with disinfectants to control HAV in bell pepper and cucumber. A high-energy (10 MeV, 30 kW) linear accelerator was used for eBeam treatment. Bell pepper and cucumber were inoculated with 8-log TCID50/mL of HAV for the experiments. eBeam treatment alone reduced HAV titers in cucumber (2.5 kGy) and bell pepper (7 kGy) by 3-log. HAV dropped below detection limits by the highest doses tested on cucumber (3 kGy) and bell pepper (10 kGy). Interestingly, treatment with a disinfectant, sodium hypochlorite (NaOCl, Sigma-Aldrich, St. Louis, MO), or chlorine dioxide (ClO_2,_ Sungchan Co., Seoul, Republic of Korea) followed by eBeam treatment resulted in reductions of more than 4.5-log using much lower eBeam doses compared to the doses required to obtain a similar HAV reduction by eBeam alone. Sequential treatment with a 50 ppm ClO_2_ and 5 kGy eBeam dose on bell pepper and a 10 ppm ClO_2_ and 1.5 kGy eBeam dose on cucumber showed the maximum efficacy for HAV reduction. Additionally, it was confirmed that the color and texture of the vegetables were unchanged even when treated with the highest eBeam dose + disinfectant combinations compared to the untreated controls. Therefore, this study certifies the efficacy of eBeam as a supplement for viral control on food while preserving the quality of the food intact.

### 3.4. Viral Inactivation for Virus Vaccine Development

EBT can also be utilized for viral inactivation and the development of anti-viral vaccines. As described in Fertey et al., 2020 [74], a mean titer of 5 × 10^5^ TCID_50_/mL influenza A (H3N8) virus was successfully inactivated by treatment with an eBeam (300 keV) dose of 22 kGy and achieved 67.3% of antigenic conservation, thereby enabling it to elicit immune responses. Furthermore, an eBeam dose of either 20 kGy or 25 kGy (200 keV) was used to inactivate a titer of 2 × 10^7^ TCID_50_/mL Respiratory Syncytial Virus (RSV) and prepare a vaccine for mice. No significant differences in antibody binding were detected between eBeam-treated RSV and live RSV (analyzed via the binding of two monoclonal antibodies, 18F12 and D25, to the F-protein), indicating the intactness of antigenic potency after eBeam treatment. Immunized mice resulted in significantly higher RSV-neutralizing antibodies compared to non-immunized mice. Additionally, 5 days after the intranasal RSV challenge to mice, it was determined that the concentration of RSV in the lungs of non-immunized mice was significantly (266-fold) higher than that of the vaccinated mice, confirming that EBT is an efficient means of generating anti-viral vaccines.

Fertey et al., 2016 [76], also describe an attempt to use EBT to inactivate high tiers of a multitude of viruses, targeting the conservation of antigenic properties and hence the retention of immunogenicity in eBeam-treated viruses. The inactivation doses used on three virus types are as follows: influenza A (H3N8)—30 kGy, Porcine Reproductive and Respiratory Syndrome Virus (PRRSV)—10 kGy, and Equine Herpesvirus 1 (EHV-1)—10 kGy (200 keV/5 mA). Upon analyzing the integrity of viral RNA for influenza A using a bioanalyzer, complete fragmentation was observed under 30 kGy. Surface antigen integrity assessment via HA assays [86] resulted in similar integrity of the eBeam-treated H3N8 surface compared to the non-irradiated control at 30 kGy, which slightly declined at 50 kGy. ELISA assays with polyclonal mice serum resulted in 90% antigen integrity of eBeam-treated H3N8, which was significantly greater than that of formalin-treated H3N8, demonstrating EBT as a better alternative with greater immunogenicity for vaccine manufacture. Polyclonal antibody-based ELISA assays with sera for PRRSV and EHV-1 also resulted in 87% and 89% antigenic integrity, respectively, after eBeam treatment. To confirm immunogenicity, female BALB/c 6–8-week-old mice were immunized with eBeam-treated H3N8, and the induction of influenza A-neutralizing antibodies was visible in the sera of this group of mice, in contradiction to that of non-immunized mice that did not develop antibodies. Intranasal H3N8 challenge resulted in only a 2% maximum weight loss in the immunized mice but a 7% maximum weight loss in the non-immunized mice. Quantitative RT-PCR assays for viral RNA in lung tissues of mice euthanized three days post-challenge resulted in average viral copy numbers of 5 × 10^4^/μL in unvaccinated control mice, 5.2 × 10/μL (982-fold significant reduction compared to control) in eBeam-treated H3N8-vaccinated mice, and up to a 382-fold reduction in formalin-treated H3N8-vaccinated mice. Though not statistically significant compared to the reduction obtained by the formalin vaccine, the eBeam vaccine induced a numerically higher reduction in viral multiplication and, therefore, greater immunity.

As raw oysters are considered a delicacy in certain countries, their consumption is a route for human exposure to human norovirus (NoV) and hepatitis A virus (HAV). Therefore, Praveen et al., 2013 [77], investigated the inactivation of HAV and MNV-1 (murine norovirus; the NoV research surrogate) in oysters. The possibility of reduction in viral multiplication in both whole oysters and oyster-meat homogenates was studied. The D_10_ values for MNV-1 in whole oysters and homogenates were 4.05 (± 0.63) and 4.97 (± 0.65) kGy, respectively, and those for HAV were 4.83 (± 0.08) and 5.74 (± 0.86) kGy, respectively. A dose of ∼23 kGy (10 MeV) resulted in 4.2-log and 4.5-log reductions in MNV-1 and HAV, respectively, and a higher dose of ∼32 kGy reduced viruses to below detectable levels. Based on the results, eBeam exposure of a standard-size serving of oysters (12 oysters at 13.68 g meat/oyster) at the maximum FDA-approved dose of 5.5 kGy at 10^5^, 10^4^, 10^3^, and 10^2^ PFU levels of HAV contamination would reduce the infection risk to humans by 16%, 39%, 74%, and 91%, respectively, compared to consumption of the same amount of non-eBeam-treated meat at the same levels of viral contamination. However, in the case of MNV-1, 12% and 26% reductions in infection per same serving size were obtained under 10^5^ PFU and 10^2^ PFU contamination conditions, respectively. Therefore, even if complete elimination may be difficult, a considerable reduction in human HAV and NoV infection following raw oyster consumption is possible via eBeam exposure, as per the conclusion of this study.

Motamedi-Sedeh et al., 2017 [78], describe an investigation of an eBeam-inactivated vaccine development to control the White Spot Syndrome Virus (WSSV) in white-leg shrimp. The D_10_ value and optimum eBeam dose (10 MeV, 2 mA) for a 10^5.4^ LD_50_/mL viral load (in post-larvae as calculated by Karber’s method [87]) were obtained as 1.85 and 13 kGy, respectively. This dose resulted in a significant viral reduction from 10^5.4^ to 10^1.5^ LD_50_/mL. The RPS (relative percentage survival [88]) values of post-larvae, when the eBeam-inactivated WSSV vaccine was administered intramuscularly and immersion route, were 73% and 75%, respectively, which were significantly higher compared to unvaccinated controls provided only with a prebiotic Immunogen (International Commerce Corporation USA, INC), which were 18.5% and 25%, respectively (0% survival in the negative control groups). Therefore, this study validates the potency of an eBeam-inactivated virus vaccine in controlling WSSV in shrimp as more efficient than prebiotics alone. Even though not significantly different compared to administering eBeam vaccination alone, the numerically highest percentage of survival was obtained when vaccination and dietary prebiotics were provided together.

Furthermore, a recent study by Skrobarczyk et al., 2022 [79], explains the efficacy of EBT as a candidate for vaccine development against human rotavirus (HRV) via its potential to generate anti-HRV IgY in immunized chicken (18-week-old, single-comb, white leghorn hens), which can be orally administered to infected humans via consumption of egg yolk. The D_10_ value was calculated as 2.38 (±0.017) kGy, and the eBeam dose needed for total inactivation (with no cytopathic effects and viral replication) was 15 kGy. According to the results of anti-VP8 chicken antiserum-based ELISAs and immunoblot assays, an 11× hyper-immunization was observed in vaccinated birds compared to unvaccinated birds. Significantly higher antigenic integrity conservation (~75–90% that of non-eBeam-exposed live virus control) was observed in the eBeam-treated HRV as opposed to formaldehyde-based and thermally inactivated HRV. Anti-HRV IgY titers ranging from 863 to 1706 in the serum and 341 to 768 in the egg yolk were observed in the post-immune state as opposed to the absence of anti-HRV IgY in pre-immune serum and egg yolk. This study also proves the almost-intact preservation of viral antigenic integrity upon exposure to EBT and its suitability to promote immunogenicity in vaccinated individuals against HRV.

A study by Liu et al., 2022 [50], has investigated the possibility of EBT controlling the spread of severe acute respiratory syndrome coronavirus 2 (SARS-CoV-2) via cold chains (low-temperature environmental conditions, i.e., 20 °C). Given the fact that cold chains are a route for SARS-CoV-2 spreading, the efficacy of high-energy eBeam (10 MeV, 2 mA) in inactivating a surrogate of SARS-CoV-2, Porcine Epidemic Diarrhea Virus (PEDV-HB3) was investigated under simulated cold-chain conditions. The virus was exposed to a dose range of 0 to 30 kGy (in increments of 5 kGy). To assess the efficacy of the eBeam-inactivated PEDV, Vero E6 cells (Institute of Microbiology, Chinese Academy of Sciences) were inoculated with untreated and eBeam-treated PEDV (exposed to various doses). No cytopathy was observed in host cells inoculated with eBeam-treated PEDV to an eBeam dose of 10 kGy or higher. An RNase assay was utilized to confirm the integrity of the viral capsid upon eBeam treatment. Here, the undamaged capsids would prevent RNase from reaching the viral RNA and subsequent degradation, where damaged capsids would result in RNA degradation by RNase. The above RNase assay followed by RT-qPCR resulted in no significant difference in the reduction in viral RNA copy numbers of 5 and 10 kGy eBeam-treated viruses, compared to the control. However, RNA copy numbers were significantly reduced in PEDVs exposed to 20 kGy or higher, implying that the capsids were significantly damaged by higher eBeam doses. Moreover, when the damage to the PEDV genome was investigated via a long-range RT-qPCR assay, the integrity of the genome upon exposure to eBeam doses of 5, 10, 20, 25, and 30 kGy was found to be significantly reduced compared to untreated PEDV by 46.25%, 62.92%, 86.63%, 92.11%, and 90.00%, respectively. This also implies that ~63% of genome damage irreversibly prevented PEDV from functioning. This study also illustrates that even though EBT is extremely versatile, it is critically important to calibrate the eBeam doses for the specific application, and generalized blanket doses should not be adopted. In conclusion, 10 kGy was identified as the optimum high-energy eBeam dose for inactivating PEDV completely (and possibly SARS-CoV-2) under frozen conditions of cold-chain environments while maintaining the integrity of the capsid, hence denoting its suitability for use as a vaccine.

Another study on RSV treatment by Eberlein et al., 2023 [80], has tested the efficacy of using low-energy electron beam-inactivated RSV (LEEI-RSV) as an alternative to the currently clinically proven interventions (passive immunization with monoclonal antibodies). The study has also tested the suitability of vaccination via the mucosal route instead of the intramuscular route, which is currently in practice. For inactivation, RSV (4.58 × 10^4^ TCID50) was exposed to 300 keV, 1.2 mA eBeam at 25 kGy. Followed by the confirmation of inactivation (observation of no cytopathic effects/CPEs) in epithelial cells (HEp-2; ATCC, Manassas, VA, USA), the eBeam-treated RSV was formulated with phosphatidylcholine (PC, LIPOID GmbH, Ludwigshafen, Germany) liposomes (PC-LEEI-RSV) for efficient delivery. Furthermore, using rodent precision cut lung slice (PCLS) technology [89,90], it was verified that the murine cells (a) exhibited no detectable toxicity (lactate dehydrogenase release) in response to incubation with 10^4^ TCID50 PC-LEEI-RSV as opposed to treatment with Triton; (b) exhibited a higher number of live cells when treated with PC-LEEI-RSV in a live/dead staining assay (LIVE/DEAD Viability/Cytotoxicity Kit, Invitrogen, Waltham, MA, USA) followed by fluorescence microscopy, when compared to treatment with live RSV and Triton; and (c) exhibited no significant difference in cytokine production between treatment with live RSV versus PC-LEEI-RSV. To test the efficacy in vivo, mice were intranasally immunized with PC-LEEI-RSV or immunized intramuscularly with LEEI-RSV adjuvanted with Alhdydrogel (LEEI-RSV i.m.) followed by a live RSV challenge. Both PC-LEEI-RSV- and LEEI-RSV i.m.-immunized mice showed significantly higher serum RSV-specific IgG profiles and lower lung RSV loads than the unvaccinated mice. Though not statistically significant, the LEEI-RSV i.m.-treated mice showed a 16-fold higher IgG titer and a 5.7-fold reduction in viral loads compared to the PC-LEEI-RSV-treated mice. In conclusion, this study validates the suitability of eBeam-treated RSV as a candidate for an anti-RSV vaccine while also portraying the efficacy of utilizing the mucosal route for vaccination.

Another study on WSSV control via EBT is reported by Sedeh et al., 2023 [82]. After having the D_10_ value for WSSV verified as 1.85 kGy, an eBeam dose of 13 kGy was used to inactivate 10(5.4) LD_50_/mL. Three vaccines [eBeam-inactivated WSSV (EBI-WSSV), eBeam-inactivated treated WSSV adjuvanted with gamma-inactivated *Vibrio parahaemolyticus* (EBI-WSSV+GIVP) and GIVP alone] and two routes of administration (injection and immersion) for all vaccines were experimented on WSSV-infected shrimp. GIVP was used as an immune stimulant. The resulting relative percentage survival (RPS) rates of shrimp from the injection-administered EBI-WSSV, EBI-WSSV+GIVP, and GIVP groups were 64%, 72%, and 22%, respectively, and the RPS rates for the same groups via immersion were 75%, 85%, and 12.5%, respectively. The RSV values for any particular group were not statistically different when the two routes were compared, but the immersion route resulted in numerically higher values. Furthermore, irrespective of the route of administration, the resulting cumulative mortalities in the eBeam-inactivated WSSV-immunized groups (both adjuvanted with GIVP and non-adjuvanted) were significantly lower compared to the group immunized with GIVP alone. Despite having numerically higher survival in the adjuvanted groups, the absence of a significant difference further proves the superiority of eBeam for viral inactivation in vaccine production. Also, it highlights the ability of an eBeam vaccine to protect without relying on an adjuvant.

### 3.5. Control of Protozoa

*Eimeria tenella* is an intracellular apicomplexan protozoan and a popular causative agent of cecal coccidiosis in chickens, causing notable losses to the poultry industry [91]. A successful attempt to control *E. tenella* in broiler chickens using low-energy electron beam (200 kV/5 mA) inactivation is explained by Thabet et al., 2019 [83]. eBeam doses of 0.1 kGy and 0.5 kGy resulted in the inhibition of in vitro reproduction of *E. tenella* by 73.2% and 86.5%, respectively. In comparison, 1 kGy or higher doses resulted in almost-complete reproductive death (assessed via a reproduction inhibition assay of *E. tenella* in Madin-Darby bovine kidney cells (DSMZ, German Collection of Microorganisms and Cell Cultures, Braunschweig, Germany)). Furthermore, D1 old chickens (12 birds/treatment) were orally inoculated with (a) Paracox^®^-8 (a commercial, live attenuated, multiple coccidia oocyst vaccine, Intervet GmbH, Germany); (b) non-irradiated *E. tenella* oocysts; (c) 0.1 kGy eBeam-exposed oocysts; and (d) 0.5 kGy eBeam-exposed oocysts. They were challenged on D21 by a single inoculation of 7.5 × 10^4^ non-attenuated oocysts. The birds were necropsied on D28 to assess the following parameters. Birds immunized with Paracox^®^-8 and 0.5 kGy eBeam-exposed *E. tenella* had higher weight gain compared to the other vaccinated groups. Non-irradiated *E. tenella*-immunized birds had significantly lower weight gain than the rest. The feed conversion ratio (FCR) was the lowest (1.76) in the negative control birds (no vaccine, no challenge), followed by 0.5 kGy eBeam-exposed *E. tenella*-vaccinated birds (1.99), while the FCR for all other groups was above 2. Coccidiosis lesion scores and oocyst index scores were significantly lower in all vaccinated chickens compared to positive control (no vaccine, *E. tenella*-challenged) birds. In vitro, reproductive inhibitions of 89.7% and 82.4% were displayed by progeny oocysts of *E. tenella* exposed to 0.5 kGy and 0.1 kGy eBeam, respectively (progeny oocysts were obtained from the feces of birds immunized with the particular group of *E. tenella*). This suggests hereditary attenuation by eBeam. Birds immunized with eBeam-treated *E. tenella* had significantly higher antibody levels than Paracox^®^-8-immunized birds. Finally, Western blot showed higher protein bands in chickens immunized with non-irradiated and 0.1 kGy eBeam-treated *E. tenella* than birds immunized with 0.5 kGy eBeam-treated *E. tenella*. Therefore, this study presents a detailed account of in vitro reproductive inhibition and in vivo protection against *E. tenella* by eBeam, as well as the importance of dose optimization for eBeam-mediated inactivation.

Eslami et al., 2020 [84], mention an attempt to use eBeam to control beef contamination with *Sarcocystis* spp., a major cause of sarcocystosis in beef. Beef samples were exposed to eBeam doses of 1, 2, 3, and 4 kGy. Twenty-four hours after eBeam treatment, relative quantification of *Sarcocystis* spp. was conducted via RNA extraction and cDNA synthesis, followed by SYBR Green RT-PCR; 3 kGy and 4 kGy eBeam-treated beef had significantly reduced *Sarcocystis* spp. compared to 0, 1, and 2 kGy eBeam-treated beef. Therefore, it was concluded that a 3 kGy eBeam dose was effective and optimal for eliminating *Sarcocystis* spp. in beef.

Apicomplexan protozoans are generally intracellular, zoonotic pathogens that infect a large range of animals, including humans [85]. Finkensieper et al., 2023 [85], explain an attempt to use low-energy eBeam to inactivate two apicomplexan protozoans: a) *Toxoplasma gondii*, a highly prevalent pathogen worldwide, responsible for virtually infecting all homeothermic animals [92] and chronically infecting one-third of the world’s population [93], and b) *Cryptosporidium parvum*, a major cause of diarrhea-associated deaths in humans [94]. In this study, a microfluidic system of low-energy eBeam treatment was used with the voltage and transportation velocity set to 200 kV and 40 mm/s, while the dose was manipulated by adjusting the current. Groups of *C. parvum* oocysts were heat-treated or treated with 0.1, 0.15, 0.2, and 2 mA eBeam (corresponding to 1.4, 2.3, 2.9, and 25 kGy doses, respectively). Excystation was halted in heat and 2 mA eBeam-treated oocysts while the excystation occurred, and the rate was not significantly different in untreated and low mA eBeam-treated oocysts. Intracellular replication (assessed via qPCR) of *C. parvum* sporozoites continued until 2 days post-inoculation of HCT-8 cells (dpi) in the non-treated control group, and the genome copy numbers of 0.1–0.2 eBeam-treated oocysts were similar to those of the control until 1 dpi but decreased thereafter. No *C. parvum* genomes were detected in the heat-treated group. *Toxoplasma gondii* tachyzoites were treated with 0.1, 0.15, and 0.25 mA eBeam (equivalent to 70, 115, and 185 Gy doses). Vero E6 cells (Eppelheim, Germany) infected with 0.1 mA exposed *T. gondii* showed infection within 3 days, but cells infected with 0.25 mA exposed *T. gondii* showed no signs of infection even after 9 dpi. Intracellular replication of *T. gondii* within Vero E6 cells (assessed by qPCR up to 10 dpi) decreased steadily up to 6 dpi in all eBeam-treated and heat-treated *T. gondii*. However, the replication of 0.1 and 0.15 mA eBeam-treated *T. gondii* increased thereafter. But the copy numbers of heat-treated and 0.25 mA eBeam-treated tachyzoites never increased (reproduction was ceased). Furthermore, in three independent experiments, no bradyzoite cysts could be found in cells infected with the eBeam-treated (at all doses) samples after 5 dpi (assessed via immunostaining). In an in vivo study, mice were immunized in three rounds with either eBeam-treated (at 0.1 or 0.25 mA) or formalin-killed *T. gondii*. By the third immunization, the 0.1 mA eBeam-treated *T. gondii*-immunized mice displayed the highest levels of anti-*T. gondii* IgG. In comparison, they were comparatively significantly lower in the 0.25 mA eBeam-treated and formalin-killed *T. gondii*-immunized mice yet significantly higher than those of the negative control group. Finally, the immunized mice were infected with a lethal strain of *T. gondii* to access the protection. All mice of the control and formaldehyde-inactivated tachyzoite-immunized groups died by the first and second weeks, respectively. Interestingly, all mice immunized with the 0.1 mA eBeam-treated tachyzoites survived for 28 dpi, while mice immunized with the 0.25 mA eBeam-treated tachyzoites showed 70% survival by 28 dpi.

## 4. Advantages of Using eBeam over Other Methods for Vaccine Development Against Microbial Pathogens

Whole-cell inactivated vaccines are considered to be advantageous over other vaccine types for treating microbial diseases for several reasons, such as their relatively lower costs and rapidness of production, the protection of susceptible antigens from acidic degradation following oral administration [95], and providing multideterminant host immunogenicity (possessing multiple immunogenic epitopes) [96]. Live whole-cell attenuated vaccines closely mirror the natural infection and hence natural host immune responses (both humoral and cellular). However, they are relatively less stable, and pathogen revival is possible. Antagonistically, inactivated whole-cell vaccines (classically treated with chemicals such as formaldehyde or other alkylating reagents [97]) are highly stable and restrict pathogen resuscitation and reproduction (hence ensuring host safety). However, they come at the cost of potentially damaging immunogenic epitopes (e.g., cross-link surface proteins), thus compromising immunogenicity (thereby necessitating the use of adjuvants to prime host immunity). Furthermore, inactivated whole-cell vaccines initiate lesser priming of CD8+ T cells and antigenic cross-presentation, which are crucial against intracellular bacteria [97,98,99,100].

Additionally, the inactivated pathogen preparation must be purified prior to vaccination (as the chemicals are also detrimental to hosts), but this is time-consuming. Alternatively, eBeam technology presents a promising solution to producing theoretically perfect whole-cell inactivated vaccines without relying on chemicals or heat by ensuring the irreversible reproductive death of eBeam-treated pathogens within the host [3], while retaining the intactness of the immunogenic epitopes of microbes upon exposure to eBeam [58,61]. Additionally, eBeam-generating equipment produces highly planar, homogenous beam profiles in high doses in a matter of seconds, facilitating efficient and rapid pathogen inactivation, as opposed to lengthy chemical-based techniques that compromise epitope integrity. Whole-cell eBeam-inactivated vaccines also provide broader protection than RNA/subunit vaccines, which carry limited epitopes. Moreover, the vaccine production process is relatively simple and less expensive than other ionizing methods (as commercial electricity is the source). Finally, eBeam technology enables the production of multi-strain microbial vaccines easily (as a mixture of microbes in a single culture can be inactivated together, given that their D_10_ values are similar). Table 5 below, summarizes the advantages of using eBeam over other strategies of vaccine development.

## 5. Challenges for the Development of eBeam-Inactivated Vaccines

The efficiency of using electron beam technology for vaccine development depends on the balance between antigenic conservation of the pathogen and the shredding of the genetic material (DNA/RNA). Simply put, the pathogen has to be inactivated entirely prior to usage as an immunostimulatory agent for vaccination by ceasing its capability for multiplication/reproduction. The inactivation is achieved by destroying their DNA/RNA during EBT with the optimum eBeam lethal dosage. A dose lower than the required one would retain the potential reproducibility of the pathogen, posing a threat to the immunized host. On the other hand, higher eBeam doses might damage cell membranes/capsids and compromise the immunogenic epitope integrity, weakening the vaccinated host’s immunogenicity and decreasing the vaccination’s efficiency. The selection of an optimum dose and complete inactivation of a considered pathogen must be carefully understood since microorganisms often have sophisticated nucleic acid repair systems. For example, during an investigation [101,102] to examine the efficiency of fractionated vs. continuous eBeam treatment on four types of viruses—Human Immunodeficiency Virus-2 (Retroviridae, enveloped HIV-2), Hepatovirus A (Picornaviridae, non-enveloped HAV), the Pseudorabies Virus (Herpesviridae, enveloped PRV), and the Porcine Parvovirus (Parvoviridae, non-enveloped PPV)—it was observed that each type of virus (except PRV) showed a slightly greater radio-resistance under continuous exposure (under multiple doses in the range of 3.4 kGy to 34 kGy) than fractionated exposure (under 3.4 Gy application up to 10 times). It was suggested that the ability of the viruses to repair sub-lethal genomic damage within the host following eBeam exposure might play a role and that fractionated exposure may hinder this process.

Moreover, in another attempt to examine the development of resistance to EBT in *E. coli* [103], the *E. coli* O157:H7 strain was inoculated in ground beef, incubated to 10^9^ CFU/g, and exposed to eBeam. Survivors were enumerated, cultured, and inoculated on ground beef for the next eBeam exposure cycle. Five consecutive cycles were conducted. It was noted that following each cycle of exposure (except for the last cycle), the D_10_ value increased significantly (from 0.24 ± 0.03 kGy in the first cycle to 0.63 ± 0.02 kGy in the fourth). Though this suggests that *E. coli* can acquire radio-resistance during exposure to eBeam, the dosimetry and the eBeam dosing performed during those studies were fraught with limitations. It is extremely critical that when such studies are performed, several experimental controls must be included to rule out experiment-induced errors and discrepancies in the results.

## 6. Future Opportunities for the Enhancement of eBeam Technology

eBeam technology has gained recognition worldwide over the past several years. There is ongoing research to improve this technology to better understand the underlying principles of microbial inactivation and how it could be harnessed for vaccine development or to improve the microbiological safety of foods. eBeam technology is one of the most robust methods of pasteurizing foods. Therefore, improvements are being directed towards developing a compact, low-energy, high-power pulsed eBeam that uses secondary electron emission, namely, the “secondary emission electron gun” (SEEG) [104]. This is compatible for use alongside a microwave (incorporated into a device like an oven, which can function on either microwave or eBeam). The eBeam mode would be a non-thermal alternative to pasteurizing fresh products (fruits, vegetables, and milk) and eliminating pathogens (for example, vegetative and spore forms of *Bacillus subtilis* and *E. coli* [105,106,107]) without cooking/degrading the quality of delicate food items. This unit requires a small and compact size, low input energy, and less shielding than conventional units of continuous, high-energy eBeam. It would be the first non-thermal antimicrobial strategy for inactivating foodborne pathogens at the point of consumption (i.e., household and food service levels) [12].

## 7. Conclusions

Ionizing radiation technology is an effective means of sterilization, pasteurization and decontamination depending on the target organism and the applied dose. Among the various types of ionizing radiation technologies, eBeam technology excels in terms of safety, cost effectivity, time conservation, and feasibility of usage as no radioactive elements are involved, no heat is generated, and its possibility to be used in a variety of environments (extending to aqueous conditions) [3,9,17]. Much industrial R&D is being conducted to improve eBeam accelerators’ reliability, compactness, and versatility.

In terms of its application in vaccine development for the control of microbial pathogens, eBeam technology excels as a most suitable alternative owing to its numerous properties. The evidence indicates that membrane and surface antigenic integrity of eBeam-exposed pathogens remain intact (even for months under lyophilized conditions [59]). Simultaneously, their cellular ATP levels and metabolic activity are conserved considerably, meaning the microbes’ activity remains except for the complete loss of reproducibility [58,60,61]. Moreover, it has also been proven that vaccinating live animals with eBeam-irradiated pathogens induces pro-inflammatory responses (TNFα) and dendritic cell maturation (MHC-II, CD40, CD80, and CD86) at levels similar to commercially used formalin-killed vaccines and certain live attenuated vaccines [59,73]. There are published reports where eBeam-treated vaccines provide significantly greater immunogenicity than commercial formaldehyde-treated vaccines [76]. Importantly, the technology is rapid, resulting in several economic and technical upsides. Thus, it is undeniable that eBeam-treated vaccines combine the safety of a “killed” vaccine with the immunogenicity of a “live attenuated” vaccine.

## Figures and Tables

**Table 2:** Brief summary of EBT studies on bacterial inactivation.

Pathogen	Host	EBT Dose	Notes	References
*S.* Montevideo	Tomato	0–2.5 kGy	Significant reduction in *S.* Montevideo when exposed to doses > 1.5 kGy.	[12,65]
*S.* Montevideo and *S.* Agona	Tomato	0–0.95 kGy	No difference in microbial inactivation between doses of 0.7 and 0.95 kGy.	[12,66]
*Salmonella*	Baby spinach	0.4 and 0.7 kGy	3.4- and 4-log reduction in rifampin-resistant *Salmonella.*	[12,67]
*S.* Tennessee and *S.* Typhimurium	Peanut butter	_	Effectively reduced and did not resuscitate over 14 days.	[12,68,69]
*S.* Enteritidis, *S.* Typhimurium, and *S.* Senftenberg	Fresh eggs	0.5 and 1.5 kGy, along with heat treatment	5-log_10_ reduction. Optimized protocol for pasteurization.	[12,70]
*S.* Typhimurium *	Chickens	2.5 kGy	3-log_10_ bacterial reduction, enhanced heterophil priming, oxidative burst, and degranulation.	[71]
*Rhodococcus equi* *	Foals	4 and 5 kGy (10 MeV)	Increased IFN-gamma production in PMBCs and increased Ig-A production. Preservation of *R. equi* cell membrane integrity.	[72]
*S.* Enteritidis *	Chickens	2.5 kGy (10 MeV, 18 kW)	Maintained a similar membrane structure to live cells and did not resuscitate in agar. Significantly lower SE colonization in the ceca, liver, spleen, and ovaries. Significantly increased serum IgG.	[61]
*Rodentibacter pneumotropicus* *	Mice	10, 20, and 30 kGy (200 keV)	100% antigenic recognition (better conservation) on eBeam treatment (time-conservative procedure) as opposed to formaldehyde inactivation (74.7%; much more time-consuming). Better LPS integrity and significantly higher IgG generation in eBeam-treated vaccinated mice. Non-severe clinical symptoms upon bacterial challenge in vaccinated groups. Significantly less bacterial colonization in the lungs of mice vaccinated with eBeam-treated bacteria compared to a formaldehyde-killed and negative control.	[73]
*Clostridium perfringens* *	Chickens	10 kGy (10 MeV, 18 kW)	Cell membrane structure, integrity, metabolic activity, and ultrastructure of the bacteria were unchanged upon exposure to eBeam. Bacterial surface antigens were retained. eBeam-treated CP did not resuscitate up to 4 weeks in an in vitro nutrient-rich conditions or in vivo, provided up to 100% protection against intestinal CP colonization and resulted in significant serum IgY increments in immunized chickens.	[58]
*Bacillus cereus* and *Escherichia coli*	(In vitro)	33 kGy for *B. cereus* and 2.2 kGy for *E. coli*. (200 keV)	Membrane structure and antigenic epitope conservation of up to 83.5% for *B. cereus* and 91.4% for *E. coli*.	[74]
*S.* Typhimurium (ST) *	Mice and in vitro (murine dendritic cells)	7 kGy (10 MeV, 15 kW)	Upon eBeam treatment, no ST resuscitation was observed for 10 days in culture media, and no colonization in mice was observed for 14 days post-inoculation. Similar metabolic activity, intact cell membranes, and similar immunoreactivity profiles (unaltered by low-temperature stress) to live ST were maintained in EBST. Increased expressions of MHC-II, CD40, CD80, CD86, and TNFα were observed in EBST-stimulated dendritic cells, and the expression levels were similar to commercial vaccines. Furthermore, the immunogenicity of the EBST vaccine was retained even after 6 months of storage under room-temperature and cold-stress conditions.	[59]
*Staphylococcus aureus* pore-forming toxin α-Hemolysin *	Mice	0, 1, 2, and 4 kGy high-energy eBeam (10 MeV/20 kW)	eBeam treatment of extracted and purified α-Hemolysin of *Staphylococcus aureus* altered its β-pleated sheet structure (the higher the dose, the higher the effect). Therefore, the ability of Hla to bind to its receptor ADAM10 and disrupt epithelial cells was significantly reduced when treated with 4 kGy, making it suitable for use as an adjuvant.	[75]

* Studies of EBT used in developing vaccinations against the corresponding pathogen. Their efficacy was tested and evaluated using animal models.

**Table 3:** Summary of EBT-mediated virus inactivation studies.

Pathogen	Host	EBT Dose	Notes	References
Influenza A (H3N8) and Respiratory Syncytial Virus (RSV) *	Mice (for RSV)	22 kGy (300 keV) for H3N8 and 20/25 kGy (200 keV) for RSV	Antigenic conservation up to 67.3% in eBeam-treated H3N8. The antigenic potency of eBeam-treated RSV is almost intact, as confirmed via monoclonal antibody binding to F-protein. Significantly higher RSV-neutralizing antibodies and significantly lower RSV loads in lung tissues were obtained in the mice immunized with the eBeam-treated RSV vaccine compared to unvaccinated mice.	[74]
Influenza A (H3N8) *, Porcine Reproductive and Respiratory Syndrome Virus (PRRSV), and Equine Herpesvirus 1 (EHV-1)	Mice	10 kGy for PRRSV and EHV-1 and 30 kGy for H3N8 (200 keV/5 mA)	Surface antigenic integrity of eBeam-treated viruses via polyclonal antibody-based ELISA was 87%, 89%, and 90% compared to that of non-irradiated controls of PRRSV, EHV-1, and H3N8, respectively. Antigenic integrity for H3N8 upon eBeam exposure was significantly greater than that following formalin inactivation procedures used in vaccine manufacture. Significantly higher antibody production and significantly lower weight loss in eBeam-treated H3N8-vaccinated mice were recorded, compared to the unvaccinated control. A 982-fold reduction in viral load in the lungs of eBeam-treated H3N8-vaccinated mice compared to a 382-fold reduction in formalin-treated H3N8-vaccinated mice.	[76]
Hepatitis A Virus (HAV) and Murine Norovirus (MNV-1)	Oysters	~23 to ~32 kGy (10 MeV)	4.2-log and 4.5-log reductions in MNV-1 and HAV on ∼23 kGy dose eBeam treatment and reduction below detectable limits on ∼32 kGy treatment. Up to 91% and 26% reductions in infection per standard serving size upon treatment with the maximum FDA-approved eBeam dose when contaminated with HAV and MNV-1, respectively.	[77]
White Spot Syndrome Virus (WSSV) *	Shrimp	13 kGy (10 MeV, 2 mA)	10^5.4^ to 10^1.5^ LD_50_/mL viral load reduction in post-larvae. Significantly increased relative percentage survival of vaccinated post-larvae (75%) compared to unvaccinated controls supplemented with prebiotics (25%) and unvaccinated post-larvae without prebiotics (0%).	[78]
Wa G1P [8] Human Rotavirus (HRV) *	Chickens	15 kGy (10 MeV, 15 kW)	The absence of viral cytopathic effects and viral replications (complete inactivation) was confirmed upon eBeam exposure at 15 kGy. ~75–90% antigenic conservation upon eBeam inactivation (significantly higher than thermal and formalin inactivation) and 11x hyper-immunization in eBeam-treated HRV-vaccinated birds. Significant increment in anti-HRV IgY in post-immune serum and the egg yolks of eBeam-treated HRV-vaccinated birds as opposed to their pre-immune state.	[79]
Porcine Epidemic Diarrhea Virus (PEDV-HB3)	(In vitro)	0–30 kGy (10 MeV, 2 mA)	A 10 kGy eBeam can effectively inactivate PEDV under frozen conditions in cold-chain environments while maintaining capsid integrity. Higher doses (>20 kGy) damaged the integrity of the capsid. Significant RNA damage was caused by eBeam of 5 kGy and above, but complete inactivation was obtained by 10 kGy or higher eBeam.	[50]
Human Respiratory Syncytial Virus (RSV) *	Mice	25 kGy (300 keV, 1.2 mA)	Confirmation of complete RSV inactivation by treatment with 25 kGy via no CPEs on epithelial cells. Confirmation of no detectable toxicity and no difference in cytokine production of eBeam-treated RSV compared to live RSV. Significantly higher serum IgG profiles and significantly lower RSV loads in RSV-challenged mice who were treated with eBeam-treated RSV.	[80]
Hepatitis A Virus (HAV)	Cucumber and bell peppers	0–3 kGy on cucumber and 0–10 kGy on bell pepper (10 MeV, 30 kW)	eBeam doses of 2.5 and 7 kGy were needed to obtain a 3-log reduction in HAV in cucumber and bell pepper, respectively. However, sequential treatment with a 50 ppm ClO_2_ and 5 kGy eBeam dose on bell pepper and a 10 ppm ClO_2_ and 1.5 kGy eBeam dose on cucumber showed the maximum efficacy for HAV reduction (above 4.5-log). The color and texture of the vegetables were unaffected.	[81]
White Spot Syndrome Virus (WSSV) *	Shrimp	D_10_ value—1.85 kGy. eBeam dose—13 kGy.	An eBeam-killed WSSV vaccine alone was compared with eBeam-killed WSSV adjuvanted with gamma-inactivated *Vibrio parahaemolyticus* via 2 routes of administration on shrimp challenged with WSSV. Both vaccines via both routes resulted in significantly higher shrimp survival compared to the control group (provided adjuvant alone). No significant difference in protection between adjuvanted and non-adjuvanted eBeam-treated WSSV, irrespective of the route of administration.	[82]

* Studies of EBT used in developing vaccinations against the corresponding pathogen. Their efficacy was tested and evaluated using animal models.

**Table 4:** Summary of EBT-mediated control of protozoans.

Pathogen	Host	EBT dose	Notes	References
*Eimeria tenella* *	Broiler chickens	0.5 and 0.1 kGy ((200 kV/5 mA)) used for the vaccine.	0.1 and 0.5 kGy low-energy eBeam treatment caused 73.2% and 86.5% reproductive death in the treated *E. tenella* oocysts and 82.4% and 89.7% reproductive death in their progeny, respectively. Immunization with eBeam-treated *E. tenella* does not affect the FCR or weight gain of broiler chickens. Coccidiosis lesions and oocyst shedding were significantly lower in eBeam-treated *E. tenella*-vaccinated followed by *E. tenella*-challenged birds than unvaccinated and unchallenged birds. Paracox^®^-8-immunized chickens had significantly lower antibody levels than eBeam-treated-*E. tenella*-vaccinated chickens.	[83]
*Sarcocystis* spp.	Beef	1, 2, 3, and 4 kGy	3 and 4 kGy eBeam significantly reduced *Sarcocystis* spp. in beef, compared to lower eBeam doses.	[84]
*Cryptosporidium parvum*	In vitro	1.4, 2.3, 2.9, and 25 kGy	Excystation was successfully prevented by heat treatment and 25 kGy eBeam treatment (but not by lower doses). Oocysts treated with 0.1–0.2 eBeam stopped replication within cells by 1 dpi.	[85]
*Toxoplasma gondii* *	In vitro and mice	70, 115, and 185 Gy	185 Gy eBeam treatment of tachyzoites completely halted in vitro replication and infection, while tachyzoites exposed to lower doses were resuscitated. All eBeam doses prevented the maturation of tachyzoites to bradyzoites. Tachyzoite-immunized mice treated with 70 Gy eBeam showed the highest levels of anti-*T. gondii* IgG and the highest survival for 28 dpi, compared to mice vaccinated with tachyzoites treated with higher eBeam doses and formalin.	[85]

* Studies of EBT used in developing vaccinations against the corresponding pathogen. Their efficacy was tested and evaluated using animal models.

**Table 5:** Summary of the advantages of using eBeam over other strategies for vaccine preparation.

Strategy	Disadvantages	Advantages of eBeam over the Strategy
Live whole-cell attenuated vaccines	Lower stability, possibility of pathogen revival.	Complete inactivation of microbes, unable to resuscitate.
Chemically inactivated whole-cell vaccines	Damage to immunogenic epitopes (protein cross-linking), lesser CD8+ T cell priming and antigenic cross-presentation, final product needs purification (labor-intensive and time-consuming).	Immunogenic epitopes are conserved (no protein cross-linking), higher priming of CD4+ and CD8+ T cells, time-conservative as purification is not needed.
RNA/subunit vaccines	Expensive, time-consuming, targets a single epitope/limited set of epitopes.	Relatively cheaper, time-conservative, provide broader protection (as the entire organism is inactivated and contained in the vaccine.)
